# A New Dental Engine

**Published:** 1874-09

**Authors:** 


### A NEW DENTAL ENGINE.

We stated in the June REGISTER that we had an intimation that there would, ere long, be presented a new and valuable dental engine. We are now able to speak more definitely upon the subject. The new engine has made its appearance from the establishment of S. S. White; and this fact is a good guarantee that it is a good thing, and from the slight examination we have made of it, we have no hesitation in expressing the opinion that it is as efficient as any engine in use. This, we doubt not, is a matter of gratification to the profession. Competition in such things is certainly desirable, for several reasons.

